# Networks of phase-amplitude neural oscillators

**DOI:** 10.1186/1471-2202-12-S1-P332

**Published:** 2011-07-18

**Authors:** Kyle CA Wedgwood, Stephen Coombes, Rüdiger Thul

**Affiliations:** 1 University of Nottingham, Nottingham, NG7 2RD, UK

## 

In mathematical descriptions of oscillating neural cells, phase reduction techniques can be used to simplify the model to a one-dimensional system [1]. This reduction allows for deeper mathematical analysis of the system, and for simulation of larger networks, since the resulting model is computationally cheaper. However, if a limit cycle is not strongly attracting then this reduction may poorly characterise behaviour of the original system when under forcing, for example, synaptic input. Here we consider a coordinate transformation to a phase-amplitude framework [2] that allows one to track the evolution of distance from the cycle as well as phase on cycle. A number of common models in computational neuroscience (including FitzHugh-Nagumo and Morris-Lecar) are revisited in this framework and their response to pulsatile current forcing is investigated. We highlight the differences between phase and phase-amplitude descriptions, and show that the former can miss some substantial features of neuronal response. Finally, we discuss extensions of this work that will allow for the description of networks of limit-cycle oscillators and improve upon the standard weakly coupled phase oscillator approach. In particular, we highlight the merits of piece-wise linear modelling for the development of a theory of strongly interacting systems.

## References

[B1] Kuramoto Y Chemical Oscillations, Waves and Turbulence 1984 Springer-Verlag

[B2] Hale JK Ordinary Differential Equations 1969 John Wiley and Sons, Inc.

